# Cerebral Near-Infrared Spectroscopy and Electrical Cardiometry During Endotracheal Suction in Ventilated Infants Following Surgery: A Feasibility Study

**DOI:** 10.3390/life15060901

**Published:** 2025-05-31

**Authors:** Matthias Nissen, Ralf-Bodo Tröbs

**Affiliations:** 1 Department of Pediatric Surgery, Marien Hospital Witten, St. Elisabeth Gruppe, Ruhr-University of Bochum, Marienplatz, D-58452 Witten, Germany; 2 Department of General-, Visceral-, and Pediatric Surgery, Section of Pediatric Surgery and Urology, St. Vinzenz Hospital, Academic Teaching Hospital of Georg-August-University Göttingen, Am Busdorf, D-33908 Paderborn, Germany; pedsurgtroe@gmail.com

**Keywords:** cerebral near-infrared-spectroscopy, electrical cardiometry, non-invasive monitoring, respiratory procedures, endotracheal suction, neonatal surgery, hemodynamics

## Abstract

**Background:** Near-Infrared Spectroscopy (NIRS) and Electrical Cardiometry (EC) are promising non-invasive techniques for monitoring tissue oxygenation and hemodynamics, particularly in surgically ill infants who struggle to maintain cerebral oxygenation and systemic perfusion. There is limited data regarding the combined use of these techniques during respiratory procedures such as endotracheal suction in intubated infants. Methods: The effects of 38 endotracheal suction maneuvers on cerebral oxygenation and cardiovascular hemodynamics were investigated in seven intubated infants following non-cardiac surgery. Parameters such as cerebral oxygenation and EC-derived metrics including heart rate, stroke volume, and cardiac output were assessed. Results: Gestational and postnatal age were 31 weeks and 16 days. During endotracheal suction, the heart rate decreased but returned to baseline afterward. After the procedure, the cerebral oxygenation, stroke volume, and cardiac output increased. Conclusions: Cerebral and systemic hemodynamics were altered during endotracheal suction maneuvers in ventilated infants. Combining NIRS and EC for monitoring cardiovascular and cerebrovascular physiology may enable more individualized therapy, helping to minimize cerebral injury in this vulnerable population.

## 1. Introduction

Mechanical ventilation (MV) is a vital intervention for critically ill infants experiencing respiratory failure [1]. MV itself may also induce cardiopulmonary adverse effects [2,3]. Among the most commonly performed procedures during MV is endotracheal suction (ETS), which is essential for removing pulmonary secretions and debris obstructing the artificial airway [4]. In preterm neonates with compromised cerebral vasoreactivity, such interventions may adversely affect cerebral hemodynamics [5]. This is particularly concerning for surgically ill and/or preterm infants, who are at an elevated risk for brain injuries and adverse neurological outcomes [6,7]. Respiratory interventions such as ETS and extubation from the endotracheal tube during MV can present challenges to the cardiovascular system, especially in the context of an underlying surgical disease that may predispose patients to hemodynamic deterioration. Various strategies have been proposed to diminish adverse effects linked to ETS. These include implementing closed suctioning systems that minimize contamination risks and maintain positive ventilation pressure, as well as adhering to evidence-based standardized protocols [4,8]. Despite these recommendations, debates around the optimal technique, timing, and decision-making for both ETS and extubation persist. The need for a careful and individualized approach remains paramount, particularly regarding the successful transition from mechanical ventilation to spontaneous breathing. Within this framework, non-invasive respiratory support systems such as high-flow nasal cannulae (HFNCs) facilitate a smoother transition to spontaneous breathing and decrease the likelihood of invasive reintubation. An individualized strategy that considers the patient’s unique clinical condition is paramount for identifying early signs of deterioration and minimizing the side effects associated with ETS and extubation [9]. In this context, emerging non-invasive techniques, including Near-Infrared Spectroscopy (NIRS) and Electrical Cardiometry (EC), present promising opportunities for individualized patient management. These tools offer continuous data on regional and systemic hemodynamics [10,11,12]. The NIRS technique employs the differential absorption spectra of near-infrared light, which are influenced by the presence of oxygenated and deoxygenated hemoglobin, to measure regional end-organ oxygenation. Studies have demonstrated acceptable agreement with weighted averages from arterial and jugular bulb oxygen saturations [13]. Conversely, EC non-invasively estimates parameters such as stroke volume (SV) and cardiac output (CO) by measuring heartbeat-dependent variations in thoracic electrical bioimpedance [14]. Thus, derived information on organ tissue oxygenation and cardiac status may be critical, particularly in high-risk neonates and infants. In contrast to NIRS, which has been evaluated in a limited number of studies regarding the effects of respiratory procedures, EC has predominantly been employed for cardiac monitoring during various clinical settings such as patent ductus arteriosus assessment, blood transfusion, patient positioning and transport, therapeutic hypothermia for hypoxic-ischemic encephalopathy, or even during neonatal thoracoscopy [14,15], with no data on ETS and limited data on extubation from MV [16,17]. Furthermore, evidence of the composite use of NIRS and EC in neonates undergoing respiratory procedures under MV is also lacking. This feasibility study utilizes both EC and NIRS during ETS maneuvers. The results indicate that cerebral oxygenation and systemic hemodynamics in ventilated infants are altered during ETS. These findings will be discussed in the current literature on utilizing EC and NIRS for monitoring during MV.

## 2. Patients and Methods

### 2.1. Patients and Design

This prospective series represents data on seven consecutive patients who underwent postoperative mechanical ventilation at a tertiary Pediatric Surgery Department over six months from June to December 2018. Continuous NIRS monitoring was performed for six patients, and EC monitoring was performed for all seven patients. The study was discontinued due to the relocation and reorganization of the Neonatal Intensive Care Unit starting in 2019. The reasons for surgery included laparotomy for necrotizing enterocolitis (three patients), incarcerated ovarian cyst (one patient), ileus following ileostomy closure (one patient), duodenal atresia (one patient), and thoracotomy for esophageal atresia repair (one patient). Inclusion criteria were parental consent, postoperative ventilation, age under one year, and the availability of a complete dataset. Exclusion criteria included patients receiving inotropic therapy at the time of measurement, the presence of hemodynamically relevant cardiac anomalies as assessed by echocardiography, severe intracranial hemorrhage, congenital heart disease, severe anemia, hypoxic-ischemic encephalitis, inherited metabolic diseases, and incomplete datasets. The estimated parameters included patients’ biometric and procedural data, cerebral regional oxygenation (c-rSO_2_), and EC-derived hemodynamic parameters such as heart rate (HR), stroke volume (SV), and cardiac output (CO) from different time points during ETS and extubation. The study was conducted when the patients were at a stable cardiorespiratory status before extubation after the reduction or discontinuation of analgosedation with Fentanyl^©^ before elective extubation. All peri-interventional maneuvers were performed or assisted by an experienced nurse under the first author’s supervision. Continuously monitored c-rSO_2_ and EC-derived data were used to calculate averages for each patient. Data were collected before (T1) and after ETS (T3) over 5-min epochs, while the duration of ETS (T2) was variable. The sample size calculation was based on the primary objective to evaluate concurrent NIRS and EC parameter utilization during ETS, as indicated by their correlation. Without existing literature on this combined application, the power analysis was drawn from a study by Gil-Anton et al. [18], which examined INVOS 5100C cerebral NIRS data with serial cardiac output measurements via femoral transpulmonary thermodilution in 15 infants. Their study showed that the systolic index positively correlated with cerebral NIRS signals (r = 0.60). Assuming similar correlation coefficients (at 80% power; alpha = 0.05; one-tailed; with 10% attrition), a sample size of 17 infants was determined necessary for this feasibility study.

### 2.2. Near-Infrared Spectroscopy

The c-rSO_2_ was measured using the INVOS 5100C^®^ device (Covidien, Mansfield, MA, USA) with left frontal hemisphere optode position (OxyAlert Neonatal NIRSensor^®^, Covidien). Before placement, the optode sites were cleaned with disinfectant wipes. The NIRS technique and its applications have been described elsewhere [10,12,19,20]. Near-infrared light is emitted at two wavelengths (730 and 810 nm), penetrating the tissue at a depth of 2–3 cm, and two photodetectors detect the reflected light placed 3 and 4 cm from the light-emitting diode. A device-specific algorithm calculates and displays the regional tissue oxygenation as the percentage of oxygen-saturated hemoglobin relative to the total hemoglobin. The raw data were collected using the INVOS Analytics^®^ tool (Version 1.2.1, Covidien) and converted to Excel^®^ for further analysis.

### 2.3. Electrical Cardiometry

The ICON^®^ device (Osypka Medical, Cardiotronic, La Jolla, CA, USA) is a non-invasive hemodynamic monitor based on the principle of transthoracic electrical bioimpedance. This technique is detailed elsewhere [21]. A low-amplitude, high-frequency electrical current is applied through the thorax, and the ohmic equivalent of mean aortic blood flow acceleration is calculated and converted into a mean aortic blood flow velocity equivalent via a device-specific algorithm. This allows for the measurement of changes in the acceleration of thoracic electrical conductivity due to the cardiac cycle-dependent realignment of erythrocytes in the aorta. Parameters such as the left ventricular stroke volume (SV) for each cardiac beat and thereof-derived cardiac output (CO), which is the product of SV over time, can be estimated [14]. The four iSense^®^ skin electrodes (Osypka Medical) were applied following the manufacturer’s instructions: one each on the left cheek, over the left carotid artery, at the left mid-axillary line at the level of the xiphoid depression, and on the lateral aspect of the left thigh [14]. Hemodynamic parameters were sampled using the iControl 4.2.1^®^ software (Osypka Medical, Cardiotronic, La Jolla, CA, USA) at five-second intervals to approximate the fixed NIRS sampling rates of 0.16–0.2 Hz. Electrical Cardiometry demonstrated precision comparable to echocardiography data in both term and preterm newborns when a signal quality index (SQI) of 80% was applied [20]. Therefore, filtering was set at 80% SQI. The collected data were transferred to an Excel^®^ sheet, and an offline review was conducted to rule out artifacts.

### 2.4. Respiratory Procedures

Volume-controlled mechanical ventilation was performed using the time-cycled, pressure-limited synchronized intermittent mandatory ventilation (SIMV) mode on the Babylog 8000 plus^®^ ventilator (Dräger Inc., Lübeck, Germany). Depending on the discretion of the anesthetist or neonatologist, various endotracheal tubes were used (see Table 1). ETS was performed under the following conditions: (i) when the ventilator indicated tube obstruction, (ii) in the presence of a sawtooth pattern on the flow-volume loop as reported by the ventilator, (iii) when oxygen saturation dropped below 90% for at least one minute after previously higher levels, (iv) if the tidal volume was 5 mL/kg or less for more than five minutes, (v) when there was an uncoordinated or asynchronous respiratory pattern with the ventilator, (vi) when secretions in or around the endotracheal tube were visible or audible, (vii) if ventilation-associated changes in blood gas values occurred, or (viii) when tachycardia, bradycardia, or agitation were present without another apparent cause. The end of the suction maneuver was defined as a restored positive pressure within the ventilation system, indicated by the return of age-adjusted tidal volume levels. ETS followed a standardized protocol per the suctioning directives of the American Association for Respiratory Care [4]. Preoxygenation was achieved by increasing the fraction of inspired oxygen by approximately 10% relative to prior levels for 30 to 60 s before suctioning and for 60 s afterward. Closed ETS was performed using a closed suction system (Halyard Health, Alpharetta, GA, USA), with a negative suction pressure of 100 mmHg. The catheter size was selected according to the manufacturer’s recommendations based on the endotracheal tube size. Suction was applied for up to 15 s with circular movements and a 60-s interval between five insertions. The catheter was advanced only as far as the end of the ETT to prevent mucosal injury and vagal reflex. While saline application was not routinely used, aliquots of 0.5 mL were employed if secretions were particularly thick. To reduce movement artifacts and their effects on circulation, all infants were kept in a supine position with the head at a 0° midline position during measurements.

### 2.5. Statistical Analysis

Data sampling and analysis were conducted using INVOS Analytics Tool^®^ Version 1.2.1 (Covidien, Mansfield, MA, USA), iControl^®^ Version 4.2.1 (Osypka Medical, Cardiotronic, La Jolla, CA, USA), Microsoft Excel^®^ (version 2010, Microsoft Corporation, Redmond, WA, USA), and OriginPro^©^ (version 2021, OriginLab, Northampton, MA, USA). Normal distribution was confirmed through the Kolmogorov–Smirnov normality test at a 0.05 significance level. Categorical variables were presented as frequencies. The median and the 1st and 3rd quartiles (Q1–Q3) were used for non-parametric data. The mean ± standard error of the mean (SEM) was utilized for parametric data. Graphical representations included box and whisker plots, illustrating the median, mean, interquartile range, and outliers. Comparative data analyses at different epochs during respiratory procedures were conducted using repeated-measures analysis of variance (ANOVA) with Tukey post-hoc testing for multiple comparisons. Mauchly’s test of sphericity was applied. Non-parametric data were compared using Friedman ANOVA with Dunn’s post hoc testing. The Kendall rank correlation coefficient (Kendall’s Τ) was employed to evaluate the relationship between the variables. A *p*-value of ≤0.05 was considered statistically significant. This study received approval from the Ethics Committee of Ruhr-University Bochum (registry no. 20-7082-BR), and written parental consent was obtained before the initiation of measurements for each case.

## 3. Results

The patients’ biometric and procedural characteristics are summarized in Table 1. The mean (±SEM) age and body weight at surgery were 36 ± 53 days and 2.2 ± 1.0 kg, respectively. Before surgery, three patients received ventilatory support: one patient was on CPAP, and two patients were intubated with SIMV ventilation. NIRS and EC recordings were obtained during 38 repeated intratracheal suction maneuvers.

While the SpO_2_ values remained unaltered, the levels of cerebral oxygenation (c-rSO_2_) were higher after ETS at T3 compared to T2 (Table 2). Compared to T1, the trending c-rSO_2_ level decreased at T2 and increased at T3. The heart rate (HR) was lower at T2 during ETS but returned to baseline levels at T3. The SV during ETS (T2) was similar to that at T1. The SV increased after ETS (T3) compared to T1, with no changes compared with T2. The CO at T3 was also higher than at T2.

**Figure 1:**
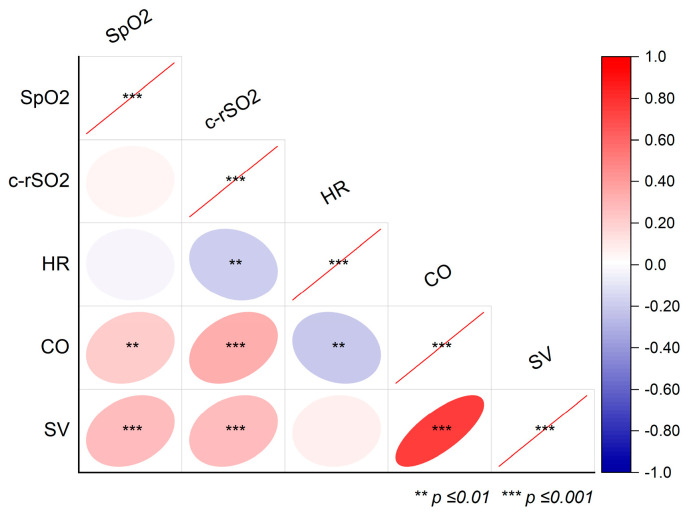
Correlations between the peripheral oxygen saturation (SpO_2_), cerebral oxygenation (c-rSO_2_), heart rate (HR), cardiac output (CO), and stroke volume (SV) during endotracheal suction. The red lines indicate correlation values of 1. The red lines indicate correlations coefficients of 1. *** is *p* ≤ 0.001.

Figure 1 illustrates the correlations between variables. Notably, the c-rSO_2_ was inversely correlated with the HR (r = −0.19, *p* = 0.008) and positively correlated with CO (r = 0.30, *p* < 0.001) and SV (r = 0.24, *p* < 0.001).

## 4. Discussion

This study aimed to assess the feasibility of combining non-invasive continuous monitoring of cerebral and systemic hemodynamics by NIRS and EC during ETS maneuvers in infants undergoing MV post-surgery.

The HR exhibited a transient decrease during ETS, subsequently returning to baseline levels. Post-ETS measurements indicated an increase in c-rSO_2_, SV, and CO. The observed trend (*p* = 0.09) toward a decline in c-rSO_2_ during ETS relative to pre-procedural values aligns with findings reported in prior studies, as summarized in Table 3 [5,22,23,24,25,26,27]. Variations in oxygenation levels during ETS have been observed in the literature, with one study reporting elevations [28] while another noted no changes [29]. In agreement with our observations, Chegondi et al. documented an increase in oxygenation following ETS [30], whereas Roll et al. reported a decrease during and after the procedure [23]. Other investigators found no alterations in oxygenation after ETS [22,31]. These discrepancies may stem from several factors, including: (a) variability in sampling time points (during vs. after ETS; duration of measurements); (b) differences in patient demographics such as gestational age (ranging from 26 to 32 weeks), postnatal age (ranging from 11.5 h to 59 months), and underlying disease (i.e., heart physiology, prematurity, etc.); (c) the deployment of multiple distinct NIRS devices across studies; (d) variations in sampling techniques and analytical algorithms [21]; and (e) heterogeneity in study design and statistical methodologies. In this context, a recent proposal has been published regarding a standardized approach to the treatment of NIRS data [32]. The observed increase in cerebral oxygenation post-ETS, coupled with the trend towards reduced oxygenation levels during ETS, warrants further investigation to elucidate whether these phenomena are causally linked to altered oxygen consumption resulting from stress-induced cortical activity or rather reflect a cumulative hemodynamic response secondary to ETS-induced sympathetic/parasympathetic activation. Drawing from existing neuroimaging research, pain processing in cortical areas may elicit hemodynamic responses, which have been quantified by functional NIRS within the prefrontal cortex [33,34,35]. In this context, the placement of the NIRS optode over the left prefrontal cortex may have allowed for the detection of fluctuations in oxygenation levels, reflecting localized cortical responses to stress or pain induced by ETS. An alternative explanation posits that hyperventilation, occurring secondarily to pain, could result in hypocapnia, inducing cerebral vasoconstriction and thus altering oxygenation levels [33]. It should be noted, however, that continuous measurements of carbon dioxide levels during ETS were not conducted, precluding the ability to rule out this confounding variable. Furthermore, in the absence of additional behavioral pain assessments, the physiological pain indicator HR in our patients was lower during ETS rather than elevated, contrary to what one might anticipate in the context of emotional stress or pain. This finding suggests that the observed bradycardia during ETS is more likely attributable to the suctioning maneuver itself and/or the release of tracheal secretions, which could trigger both sympathetic and parasympathetic activation. Most previous studies have documented reductions in HR during ETS [22,23,24,25,26], likely due to vagal reflexes elicited by ETS or alterations in intrathoracic pressure related to changes in airway dynamics or patient responsiveness. The procedure of ETS may also result in reduced peripheral oxygen saturation, either directly due to the maneuver or resulting from possible endotracheal tube obstruction, thereby underscoring the necessity for ETS. The existing literature on ETS and NIRS has frequently documented decreases in oxygen saturation during the maneuver [5,22,23,24,25,26,28,29], showing varying degrees of oxygen saturation recovery thereafter [22,29,30,31] (Table 3). In contrast, as oxygen saturation remained stable throughout our observations, with notable alterations in HR and cerebral oxygenation, this may suggest that NIRS is a more sensitive monitoring modality compared to the conventional SpO_2_ assessment; however, further elucidation is necessary to draw definitive conclusions. The observed increases in SV and CO post-ETS might be attributable to changes in intrathoracic dynamics (i.e., an enhanced pulmonary functional vital capacity) and associated cardiovascular parameters (i.e., increased preload) or potentially might be reflective of emotional arousal or pain; determining the causality remains elusive at this juncture. To this date, a combined assessment of EC and NIRS in surgically ill children, or during respiratory interventions under MV, has not been conducted. When considering the findings from both monitoring techniques, the concordance of increased NIRS levels alongside elevated CO and SV suggests a potential correlation with enhanced systemic hemodynamics, which is supported by our finding of positive correlations between cerebral oxygenation and both CO and SV (Figure 1). Research has identified significant correlations between NIRS and conventional thermodilution-derived cardiac parameters. For instance, Lanning et al. demonstrated that alterations in cerebral NIRS are indicative of changes in cardiac index during adult cardiac surgery [36]. Similarly, Gil-Anton et al. reported that the combined assessment of cerebral and renal NIRS correlates with CO and is capable of detecting events of low CO in pediatric patients following congenital heart surgery, suggesting that NIRS may serve as a good estimate for monitoring CO in this cohort [18]. Consequently, NIRS might not only provide valuable insights into regional oxygenation status but may also comprise a reference metric for the precise calibration of EC-derived parameters such as CO. In the future, a comprehensive assessment that integrates NIRS data could enhance the differentiation of various clinical conditions and individual patient scenarios, potentially augmented through the implementation of artificial intelligence-driven algorithms to optimize data interpretation and monitoring strategies. Notable composite assessments of NIRS and EC include a study by Truong et al. on the effects of premedication before intubation in neonates [37], another by Martini et al. examining cardio- and cerebrovascular responses in preterm infants during transitional phases [38], and Forman et al.’s investigation into cardiac output and cerebral perfusion monitoring in infants with encephalopathy [39].

In general, there is a paucity of knowledge regarding the influence of these biophysical changes on clinical outcomes. The American Heart Association has observed that, despite the increasing adoption of Near-Infrared Spectroscopy (NIRS), the evidence linking its use to improved neurological outcomes in pediatric cardiac populations remains limited and occasionally contradictory [40] Hansen et al. conducted a systematic review and meta-analysis on cerebral oxygenation monitoring via NIRS, concluding that there were no significant differences in mortality or neurological outcomes between groups with and without NIRS monitoring [41]. Wang et al. explored the clinical significance of the disturbance coefficient (an electrical bioimpedance parameter) and regional cerebral NIRS in pediatric neurocritical care, identifying potential thresholds for brain edema and poor prognosis [42]. Loo et al. systematically reviewed the effectiveness of NIRS, EEG, and biochemical biomarkers in predicting neurodevelopmental outcomes following pediatric congenital heart disease surgery, finding limited evidence for NIRS but promising results for EEG and lactate levels [43]. Pardo et al. conducted a scoping review on neuromonitoring methods for neonates with congenital heart disease, emphasizing the potential of various techniques for predicting outcomes while acknowledging limitations and suggesting areas for future research [44].

The primary limitation of this feasibility study is its low statistical power, attributable to the study’s discontinuation due to unforeseen administrative issues, which prevented the achievement of the predefined sample size of 19 patients. With only seven patients enrolled, the results cannot be considered conclusive. It is necessary to replicate the study with a larger sample size to draw definitive conclusions. Such a study may potentially elucidate the advantages of integrating multimodal biomarkers from these two non-invasive digital monitoring systems that reflect systemic and cerebral hemodynamics. Further study limitations include the single-center design and the lack of transcutaneous blood gas monitoring during ETS, which could have provided information on a potential link between CO_2_-dependent c-rSO_2_ variability.

In conclusion, our study demonstrated the feasibility of using NIRS and EC simultaneously to detect changes in cerebral and systemic hemodynamics during endotracheal suction in mechanically ventilated infants post-surgery. The preliminary results are consistent with established physiological responses; however, definitive biological implications cannot be determined at this stage. Generally, the lack of standardized study designs and the variability in devices impede the comparability of research findings in this field. Although the study was discontinued due to the relocation of our intensive care unit, our preliminary findings on the combined assessment of NIRS and EC highlight the potential of these techniques for individualized monitoring, particularly for surgically ill infants, to mitigate cerebral injury in this vulnerable population. Further research is essential to refine these technologies and elucidate the clinical relevance of these multi-component non-invasive digital biomarkers.

## Figures and Tables

**Table 1:** Basic biometric and procedural characteristics.

Patient’s ID	01	02	03	04	05	06	07	Σ
Gender (n; f/m)	f	f	f	m	m	m	f	4/3
Gestational age (weeks)	34	26	42	28	31	26	33	31 (26–42)
Birth weight (kg)	1.94	0.40	3.45	1.18	1.70	0.71	1.08	1.2 (0.4–3.5)
Postnatal age (d)	6	149	1	34	16	44	1	16 (1–149)
Weight (kg)	1.94	3.79	3.1	1.95	2.14	1.38	1.08	2.0 (1.1–3.8)
Length (cm)	46	48	55	45	41	37	36	44 (36–55)
Type of surgery	Lap/iOC	Lap/ileus *	Tho/EA	Lap/NEC	Lap/NEC	Lap/NEC	Lap/DA	
Comorbidities	-	BPD, PVL	VSD	BPD	-	MMC	VSD	
Respiratory data								
ETT type	Vygon	Portex	Vygon	Vigon	Rüsch	Vigon	Portex	
ETT inner diameter (mm)	3.0	3.5	3.0	2.5	3.0	2.5	2.5	
Vent. support before surgery (y/n) / type	n	n	n	y/CPAP	y/SIMV	y/SIMV	n	
ET-intubation time (hrs)	26	13.5	56	65	150	506	94	77 (13.5–506)

Lap—laparotomy; Tho—thoracotomy; iOC—incarcerated ovarian cyst; *—following ileostomy closure after midgut volvulus; EA—esophageal atresia (Vogt 3b); NEC—necrotizing enterocolitis; DA—duodenal atresia; BPD—bronchopulmonary dysplasia; PVL—periventricular leukomalacia; VSD—ventricular septum defect (both hemodynamically non-significant VSD with normal stroke volume on echocardiography); MMC—meningomyelocele; ETT—endotracheal tube; Vent.—ventilatory CPAP continuous positive airway pressure; HFNC—high-flow nasal cannula; NIRS—Near-Infrared Spectroscopy, EC—Electrical Cardiometry; SIMV—synchronized intermittent mandatory ventilation. * Summarized data are expressed as median (min.–max.).

**Table 2:** NIRS and EC-derived hemodynamic data from 38 endotracheal suction events in ventilated infants.

	Endotracheal Suction			Overall Model Characteristics	Pairwise Post Hoc Testing (*p*-Values)		
	T1 (before)	T2 (during)	T3 (after)		T1 vs. T2	T1 vs. T3	T2 vs. T3
SpO_2_ (%)	97 (95–99)	96 (95–99)	97 (95–99)	x^2^ (2) = 0.55 (*p* = 0.76)	1	1	1
HR (1/min)	144 ± 3	136 ± 3	142 ± 2	F (2,68) = 7.87 (*p* < 0.001)	**<0.001**	0.39	**0.032**
c-rSO_2_ (%)	76 (68–81)	75 (65–81)	79 (70–82)	x^2^ (2) = 19.1 (*p* < 0.001)	0.09	0.09	**<0.001**
SV (mL/beat)	3.3 (2.7–4.1)	3.4 (3–4.2)	3.5 (3.2–4.5)	x^2^ (2) = 10.5 (*p* = 0.005)	0.46	**0.004**	0.22
CO (L/min)	0.48 (0.43–0.57)	0.48 (0.38–0.56)	0.5 (0.45–0.58)	x^2^ (2) = 8.9 (*p* < 0.013)	0.70	0.25	**0.01**

T1–3—timepoints 1–3; SpO_2_—peripheral capillary oxygen saturation_;_ c-rSO_2_—cerebral regional oxygenation_;_ HR—heart rate; SV—stroke volume; CO—cardiac output. Data are presented as mean ± SEM or as median (Q1–Q3). Statistical differences are given in bold.

**Table 3:** The literature on NIRS and EC during endotracheal suction in ventilated children.

Reference	Present Study	Merter et al. [22]	Misirlioglu et al. [29]	Chegondi et al. [30]	Limperopoulos et al. [28]	Roll et al. [23]	Kohlhauser et al. [24]	Bernert et al. [25]	Mosca et al. [31]	Skov et al. [5]	Shah et al. [26]	Bucher et al. [27]
Study year	2025	2024	2022	2017	2008	2001	2000	1997	1997	1992	1992	1989
Patients (n)	6	51	20	19	82	12	15	13	11	29	12	11
ETS events (n)	38	51	70	287	96	12	26	20	66	58	≤36	n.s.
GA (weeks)	31	32	n.a.	n.a.	26	28	28.5	30	29	31	30	*“prematures”*
Postnatal age	16 d	5 d	59 m	5.7 y	11.5 h	12 h	9.7/4.6 d *	2 d	3 d	36 h	11 d	n.s.
NIRS Device	Invos 5100C	Invos 5100C	Invos 5100C	INVOS (n.s.)	NIRO-500	Kritikon Redox 2020	NIRO-500	NIR1000	Niro-500	NIR (n.s.)	Linear Instruments	NIR1000
Mean arterial pressure	n.a.	n.a.	d&a↔	a↑	d↔	d&a↔	d↔	d↑(*n* = 5), ↓(*n* = 3), ↔(*n* = 12)	a↔	d↔	d↑	n.a.
Heart rate	d↓, a↔	d↓, a↔	d↑, a ↔	a↓	n.a.	d↓	d↓	d↓	a↓	n.a.	d↓	n.a.
Oxygen saturation	d&a↔	d↓, a↑	d↓, a↔	a↓	d↓	d↓	d↓	d↓	a↓	d↓	d↓	n.a.
Cerebral NIRS	d↓(trend), a↑	d↓, a↔	d↔	a↑	d↑	d&a↓	d↓	d↓	a↔	d↓	d↓	d↓
Stroke volume	d↔, a↑	-	-	-	-	-	-	-	-	-	-	-
Cardiac output	d↔, a↑	-	-	-	-	-	-	-	-	-	-	-

d—during; a—after; ↓—decrease; ↑—increase; ↔—unchanged (relative to pre-suction level); m—months; y—years; d—days; h—hours; *—CV/HFOV conventional ventilation (CV) in six infants (ten events)/high-frequency oscillation ventilation (HFOV) in nine infants (16 events); NIRS—Near-Infrared Spectroscopy; n.a.—not available; n.s.—not specified.

## Data Availability

The raw data supporting the conclusions of this article will be made available by the corresponding author, M.N., without undue reservation.

## Abbreviations

The following abbreviations are used in this manuscript:

ANOVA	Analysis of variance
CO	Cardiac output
CPAP	Continuous positive airway pressure
c-rSO_2_	Cerebral regional oxygenation
EC	Electrical Cardiometry
ETS	Endotracheal suction
HFNC	High-flow nasal cannula
HR	Heart rate
Hz	Hertz
MV	Mechanical ventilation
NIRS	Near-Infrared Spectroscopy
SIMV	Synchronized intermittent mandatory ventilation
SpO_2_	Peripheral oxygen saturation
SQI	Signal quality index
SV	Stroke volume
T1–3	Timepoints 1–3 (before, during, and after ETS)

## References

[1] Chakkarapani A.A., Adappa R., Ali S.K.M., Gupta S., Soni N.B., Chicoine L., Hummler H.D. (2020). “Current Concepts of Mechanical Ventilation in Neonates”—Part 1. Int. J. Pediatr. Adolesc. Med..

[2] Shekerdemian L., Bohn D. (1999). Cardiovascular Effects of Mechanical Ventilation. Arch. Dis. Child..

[3] Sehgal A., Ruoss J.L., Stanford A.H., Lakshminrusimha S., McNamara P.J. (2022). Hemodynamic Consequences of Respiratory Interventions in Preterm Infants. J. Perinatol..

[4] American Association for Respiratory Care (2010). AARC Clinical Practice Guidelines. Endotracheal Suctioning of Mechanically Ventilated Patients with Artificial Airways 2010. Respir. Care.

[5] Skov L., Ryding J., Pryds O., Greisen G. (1992). Changes in Cerebral Oxygenation and Cerebral Blood Volume during Endotracheal Suctioning in Ventilated Neonates. Acta Paediatr..

[6] Stolwijk L.J., Keunen K., de Vries L.S., Groenendaal F., van der Zee D.C., van Herwaarden M.Y.A., Lemmers P.M.A., Benders M.J.N.L. (2017). Neonatal Surgery for Noncardiac Congenital Anomalies: Neonates at Risk of Brain Injury. J. Pediatr..

[7] Selvanathan T., Au-Young S.H., Guo T., Chau V., Branson H.M., Synnes A., Ly L., Kelly E.N., Grunau R.E., Miller S.P. (2023). Major Surgery, Brain Injury, and Neurodevelopmental Outcomes in Very Preterm Infants. Neurology.

[8] Gardner D., Shirland L. (2009). Evidence-Based Guideline for Suctioning the Intubated Neonate and Infant. Neonatal Netw..

[9] Clifton-Koeppel R. (2006). Endotracheal Tube Suctioning in the Newborn: A Review of the Literature. Newborn Infant. Nurs. Rev..

[10] Kooi E.M.W., Mintzer J.P., Rhee C.J., Ergenekon E., Schwarz C.E., Pichler G., de Boode W.P., Alarcón A., Alderliesten T., Austin T. (2024). Neonatal Somatic Oxygenation and Perfusion Assessment Using Near-Infrared Spectroscopy. Pediatr. Res..

[11] Pellicer A., de Boode W., Dempsey E., Greisen G., Mintzer J., Naulaers G., Pichler G., Roehr C.C., Roll C., Schwarz C. (2024). Cerebral Near-Infrared Spectroscopy Guided Neonatal Intensive Care Management for the Preterm Infant. Pediatr. Res..

[12] Levy P.T., Pellicer A., Schwarz C.E., Neunhoeffer F., Schuhmann M.U., Breindahl M., Fumagelli M., Mintzer J., de Boode W., Alarcon A. (2024). Near-Infrared Spectroscopy for Perioperative Assessment and Neonatal Interventions. Pediatr. Res..

[13] Nasr V.G., Bergersen L.T., Lin H.-M., Benni P.B., Bernier R.S., Anderson M.E., Kussman B.D. (2019). Validation of a Second-Generation Near-Infrared Spectroscopy Monitor in Children with Congenital Heart Disease. Anesth. Analg..

[14] O’Neill R., Dempsey E.M., Garvey A.A., Schwarz C.E. (2021). Non-Invasive Cardiac Output Monitoring in Neonates. Front. Pediatr..

[15] Soaida S., Hanna M., Mahmoud A., Elmetwally S. (2021). Electrical Velocimetry (ICON Cardiometry) Assessment of Hemodynamic Changes Associated with Different Inflation Pressures during Pediatric Thoracoscopic Surgery: A Pilot Study. Egypt. J. Cardiothorac. Anesth..

[16] Rachel M., Jan M., Heather C., Jana S. (2022). Non-Invasive Cardiac Output Monitoring before and after Baby Extubation—A Feasibility Study (NICOMBabe Study). Early Hum. Dev..

[17] Boet A., Jourdain G., Capderou A., Grollmuss O., Labrune P., De Luca D., Demontoux S. (2014). PO-0488 Non-Invasive Haemodynamic Monitoring Using Electrical Cardiometry in Neonates During Respiratory Procedures. Arch. Dis. Child..

[18] Gil-Anton J., Redondo S., Urabayen D.G., Faza M.N., Sanz I., Pilar J. (2015). Combined Cerebral and Renal Near-Infrared Spectroscopy After Congenital Heart Surgery. Pediatr. Cardiol..

[19] Marin T., Moore J. (2011). Understanding Near-Infrared Spectroscopy. Adv. Neonatal Care.

[20] Noori S., Drabu B., Soleymani S., Seri I. (2012). Continuous Non-Invasive Cardiac Output Measurements in the Neonate by Electrical Velocimetry: A Comparison with Echocardiography. Arch. Dis. Child. Fetal Neonatal Ed..

[21] van Wyk L., Austin T., Barzilay B., Bravo M.C., Breindahl M., Czernik C., Dempsey E., de Boode W.-P., de Vries W., Eriksen B.H. (2024). A Recommendation for the Use of Electrical Biosensing Technology in Neonatology. Pediatr. Res..

[22] Merter O.S., Dertli S., Taskin E., Aydin M., Benli S. (2024). Effects of Endotracheal Suctioning Duration Cerebral Oxygenation in Preterm Infants. J. Clin. Nurs..

[23] Roll C., Horsch S., Knief J., Hüsing J., Hanssler L. (2001). Vergleich Der Effekte von Endotrachealem Absaugen Und Surfactantapplikation Auf Hämodynamik Und Oxygenierung Frühgeborener—Eine Nahinfrarotspektroskopie-Studie1. Z. Geburtshilfe Neonatol..

[24] Kohlhauser C., Bernert G., Hermon M., Popow C., Seidl R., Pollak A. (2000). Effects of Endotracheal Suctioning in High-Frequency Oscillatory and Conventionally Ventilated Low Birth Weight Neonates on Cerebral Hemodynamics Observed by near Infrared Spectroscopy (NIRS). Pediatr. Pulmonol..

[25] Bernert G., von Siebenthal K., Seidl R., Vanhole C., Devlieger H., Casaer P. (1997). The Effect of Behavioural States on Cerebral Oxygenation during Endotracheal Suctioning of Preterm Babies. Neuropediatrics.

[26] Shah A.R., Kurth C.D., Gwiazdowski S.G., Chance B., Delivoria-Papadopoulos M. (1992). Fluctuations in Cerebral Oxygenation and Blood Volume during Endotracheal Suctioning in Premature Infants. J. Pediatr..

[27] Bucher H.U., Blum-Gisler M., Duc G. (1993). Changes in Cerebral Blood Volume during Endotracheal Suctioning. J. Pediatr..

[28] Limperopoulos C., Gauvreau K.K., O’Leary H., Moore M., Bassan H., Eichenwald E.C., Soul J.S., Ringer S.A., Di Salvo D.N., du Plessis A.J. (2008). Cerebral Hemodynamic Changes During Intensive Care of Preterm Infants. Pediatrics.

[29] Misirlioglu M., Horoz O.O., Yildizdas D., Ekinci F., Yontem A., Menemencioglu A., Salva G. (2022). The Effects of Endotracheal Suctioning on Hemodynamic Parameters and Tissue Oxygenation in Pediatric Intensive Care Unit. J. Pediatr. Intensive Care.

[30] Chegondi M., Francis T., Lin W.-C., Naqvi S., Raszynski A., Totapally B.R. (2018). Effects of Closed Endotracheal Suctioning on Systemic and Cerebral Oxygenation and Hemodynamics in Children. Pediatr. Crit. Care Med..

[31] Mosca F.A., Colnaghi M., Lattanzio M., Bray M., Pugliese S., Fumagalli M. (1997). Closed versus Open Endotracheal Suctioning in Preterm Infants: Effects on Cerebral Oxygenation and Blood Volume. Biol. Neonate.

[32] Vesoulis Z.A., Mintzer J.P., Chock V.Y. (2021). Neonatal NIRS Monitoring: Recommendations for Data Capture and Review of Analytics. J. Perinatol..

[33] Sakuma S., Inamoto K., Higuchi N., Ariji Y., Nakayama M., Izumi M. (2014). Experimental Pain in the Gingiva and its Impact on Prefrontal Cortical Hemodynamics: A Functional near-Infrared Spectroscopy Study. Neurosci. Lett..

[34] Yücel M.A., Aasted C.M., Petkov M.P., Borsook D., Boas D.A., Becerra L. (2015). Specificity of Hemodynamic Brain Responses to Painful Stimuli: A Functional Near-Infrared Spectroscopy Study. Sci. Rep..

[35] Becerra L., Aasted C.M., Boas D.A., George E., Yücel M.A., Kussman B.D., Kelsey P., Borsook D. (2016). Brain Measures of Nociception Using Near-Infrared Spectroscopy in Patients Undergoing Routine Screening Colonoscopy. Pain.

[36] Lanning K.M., Ylikauma L.A., Erkinaro T.M., Ohtonen P.P., Vakkala M.A., Kaakinen T.I. (2023). Changes in Transcranial Near-Infrared Spectroscopy Values Reflect Changes in Cardiac Index during Cardiac Surgery. Acta Anaesthesiol. Scand..

[37] Truong L., Kim J.H., Katheria A.C., Finer N.N., Marc-Aurele K. (2020). Haemodynamic Effects of Premedication for Neonatal Intubation: An Observational Study. Arch. Dis. Child. Fetal Neonatal Ed..

[38] Martini S., Frabboni G., Rucci P., Czosnyka M., Smielewski P., Galletti S., Cimatti A.G., Faldella G., Corvaglia L., Austin T. (2020). Cardiovascular and Cerebrovascular Responses to Cardio-Respiratory Events in Preterm Infants during the Transitional Period. J. Physiol..

[39] Forman E., Breatnach C.R., Ryan S., Semberova J., Miletin J., Foran A., EL-Khuffash A. (2017). Noninvasive Continuous Cardiac Output and Cerebral Perfusion Monitoring in Term Infants with Neonatal Encephalopathy: Assessment of Feasibility and Reliability. Pediatr. Res..

[40] Marino B.S., Tabbutt S., MacLaren G., Hazinski M.F., Adatia I., Atkins D.L., Checchia P.A., DeCaen A., Fink E.L., Hoffman G.M. (2018). Cardiopulmonary Resuscitation in Infants and Children with Cardiac Disease. Circulation.

[41] Hansen M.L., Hyttel-Sørensen S., Jakobsen J.C., Gluud C., Kooi E.M.W., Mintzer J., de Boode W.P., Fumagalli M., Alarcon A., Alderliesten T. (2024). Cerebral Near-Infrared Spectroscopy Monitoring (NIRS) in Children and Adults: A Systematic Review with Meta-Analysis. Pediatr Res.

[42] Wang C., Xing D., Zhou S., Fang F., Fu Y., Xu F. (2023). Electrical Bioimpedance Measurement and Near-Infrared Spectroscopy in Pediatric Postoperative Neurocritical Care: A Prospective Observational Study. Front Neurol.

[43] Van Loo L., Cools B., Dereymaeker A., Jansen K. (2024). Neuromonitoring Modalities Predicting Neurological Impairment in Pediatric Congenital Heart Disease: A Systematic Review. Front Neurol.

[44] Pardo A.C., Carrasco M., Wintermark P., Nunes D., Chock V.Y., Sen S., Wusthoff C.J., Bonifacio S., Aly H., Chau V. (2024). Neuromonitoring Practices for Neonates with Congenital Heart Disease: A Scoping Review. Pediatr Res.

